# Crystal structures of 4-[(4-methyl­benz­yl)­oxy]benzohydrazide and its *N*′-[(thio­phen-2-yl)­methyl­idene]- derivative

**DOI:** 10.1107/S2056989023001354

**Published:** 2023-02-21

**Authors:** Md. Hasan Al Banna, Md. Belayet Hossain Howlader, Ryuta Miyatake, Md. Chanmiya Sheikh, Md. Rezaul Haque Ansary, Ennio Zangrando

**Affiliations:** a Department of Chemistry, Rajshahi University, Rajshahi-6205, Bangladesh; b Center for Environmental Conservation and Research Safety, University of Toyama, 3190 Gofuku, Toyama, 930-8555, Japan; c Department of Applied Science, Faculty of Science, Okayama University of Science, Japan; d Department of Chemical and Pharmaceutical Sciences, University of Trieste, Italy; Vienna University of Technology, Austria

**Keywords:** crystal structure, hydrazine, hydrazone

## Abstract

The mol­ecular and crystal structures of a benzoyl­hydrazine bearing an ether group, 4-[(4-methyl­benz­yl)­oxy]benzohydrazide, C_15_H_16_N_2_O_2_, (I), and of the corresponding *N*′-[(thio­phen-2-yl)­methyl­idene]- derivative, 4-[(4-methyl­benz­yl)­oxy]-*N*′-[(thio­phen-2-yl)­methyl­idene]benzohydrazide, C_20_H_18_N_2_O_2_S, (II), are described. The supra­molecular structures of both compounds are governed by N—H⋯N and N—H⋯O hydrogen-bonding inter­actions. The hydrazine compound (I) shows a crystal packing with a more complex hydrogen-bonding scheme because of the NH—NH_2_ entity, forming a di-periodic supra­molecular structure extending parallel to (100). Hydrazone mol­ecules in (II) are hydrogen-bonded through N—H⋯O inter­actions, giving rise to the formation of ribbons parallel to [010]. Mol­ecules of (I) and (II) show a different orientation of the carbohydrazide moiety likely to favor the crystal packing and thus hydrogen-bonding inter­actions.

## Abstract 2

The crystal and mol­ecular structures of a benzoyl­hydrazine bearing an ether group and of its *N*′-[(thio­phen-2-yl)­methyl­idene derivative are reported and compared.

## 1. Chemical context

Hydrazine-based compounds occupy a prominent position in chemistry (Sandler & Karo, 1992 ▸) because of their pharmaceutical uses (Popiołek, 2021 ▸) and many other applications (Mali *et al.*, 2021 ▸; Koz’minykh, 2006 ▸). Their increasing importance originates from anti-inflammatory (Todeschini *et al.*, 1998 ▸), anti­bacterial–anti­fungal (Vicini *et al.*, 2002 ▸), and anti­tubercular (Bedia *et al.*, 2006 ▸) properties, as well as their utilization as pesticides (Pandey *et al.*, 2020 ▸). However, it is worth noting that hydrazine-based compounds applied as rocket fuels pose significant health risks owing to their toxicity (Sinha & Mason, 2014 ▸). In addition, hydrazine-based compounds function as ligand precursors for the formation of bidentate Schiff base ligands applied in metal coordination (Banna *et al.*, 2022 ▸; Zhou *et al.*, 2006 ▸; Alagesan *et al.*, 2013 ▸; Chen *et al.*, 2022 ▸).

In the context given above, we report on syntheses and crystal-structure determinations of two related compounds, *viz*. a benzoyl­hydrazine bearing an ether group (I), C_15_H_16_N_2_O_2_, and the corresponding *N*′-[(thio­phen-2-yl­meth­yl­idene) derivative (II), C_20_H_18_N_2_O_2_S.

## 2. Structural commentary

**Figure 1:**
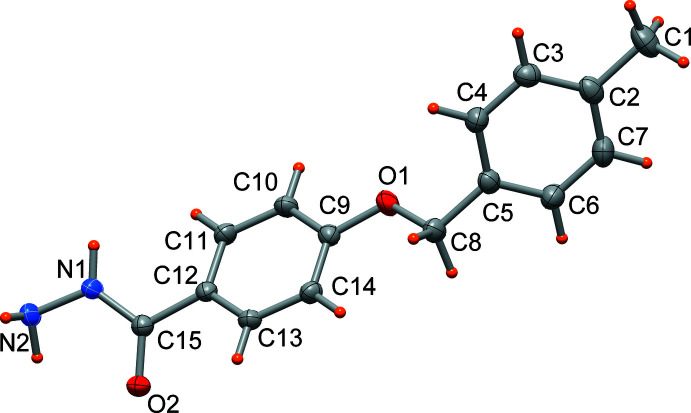
Mol­ecular structure of (I), with displacement ellipsoids drawn at the 50% probability level.

The mol­ecular structure of hydrazine compound (I) is shown in Fig. 1 ▸. The N1—N2 and the O2=C15 bond lengths of 1.4200 (15) and 1.2388 (15) Å are indicative of a single and double bond, respectively. All other bond lengths are as expected when compared with mol­ecules of similar hydrazine and hydrazone compounds (Wang, Zhou *et al.*, 2014 ▸; Wang, He *et al.*, 2014 ▸; Fun *et al.*, 2012 ▸; Zong & Wu, 2013 ▸). The conformation of the mol­ecule shows the central phenyl ring (C9–C14) of the benzoyl mean plane forming a dihedral angle of 66.39 (3)° with the 4-methyl­benzyl group (C1–C8), and it is also rotated slightly [by 28.49 (6)°] with respect to the mean plane through the C=O—NH—NH_2_ moiety. 
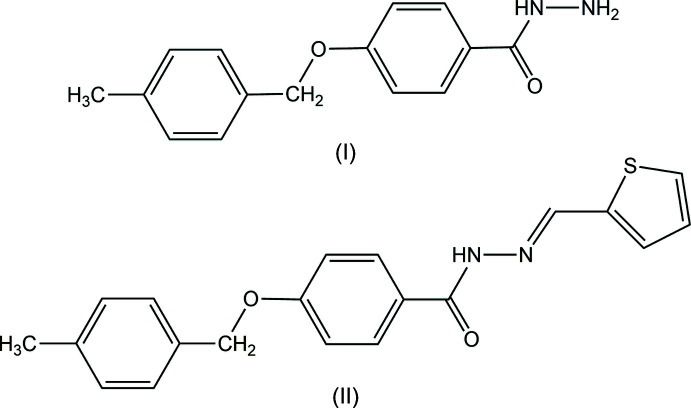


**Figure 2:**
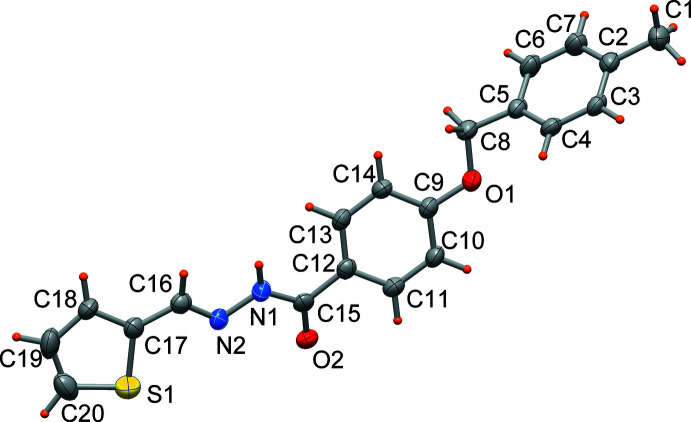
Mol­ecular structure of (II), with displacement ellipsoids drawn at the 50% probability level.

The mol­ecular structure of hydrazone derivative (II) is shown in Fig. 2 ▸. The thienyl (C17–C20, S1) ring and the central phenyl ring (C9–C14) are linked by the ac­yl–hydrazone (–CH=N—N—CO–) group. An *E*-configuration is observed with respect to the double bond of the hydrazone bridge N2=C16. Compared to (I), the N1—N2 bond length of 1.397 (4) Å appears slightly shorter, most probably caused by a different inter­molecular hydrogen-bonding inter­action. On the other hand, the O2=C15 bond of 1.236 (4) Å, is nearly identical with that of (I) and is typical for a ketonic linkage in the solid state, while an equilibrium between the keto and enol form is present in solution. The mol­ecule has the thienyl­methyl­ene and the benzohydrazone fragments almost co-planar, with maximum deviations of −0.234 (3) and +0.392 (2) Å exhibited by atoms C10 and O2, respectively. The terminal 4-methyl­benzyl group is rotated by 55.87 (9)° with respect to the central phenyl ring, similar to the dihedral angle observed in (I).

**Figure 3:**
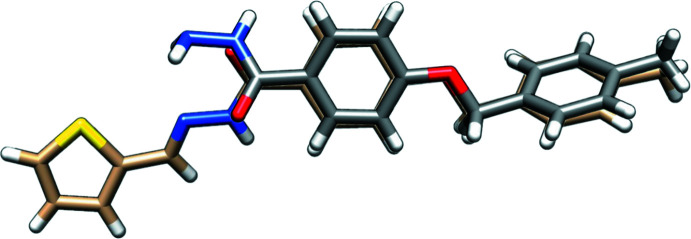
Overlay plot of mol­ecules (I) and (II) to show the conformational difference.

A superimposition of the two mol­ecules (shown in Fig. 3 ▸) highlights their conformational differences: while the 4-[(4-methyl­benz­yl)­oxy] benzoyl groups almost overlap, it is worthy to note the different orientation of the carbohydrazide C=O—NH—N moieties, likely induced by crystal packing effects to favor hydrogen-bonding inter­actions.

## 3. Supra­molecular features

Classical hydrogen-bonding inter­actions represent the main contributions to the packing of the mol­ecules in the crystals of (I) and (II); numerical data are compiled in Tables 1 ▸ and 2 ▸, respectively.

**Figure 4:**
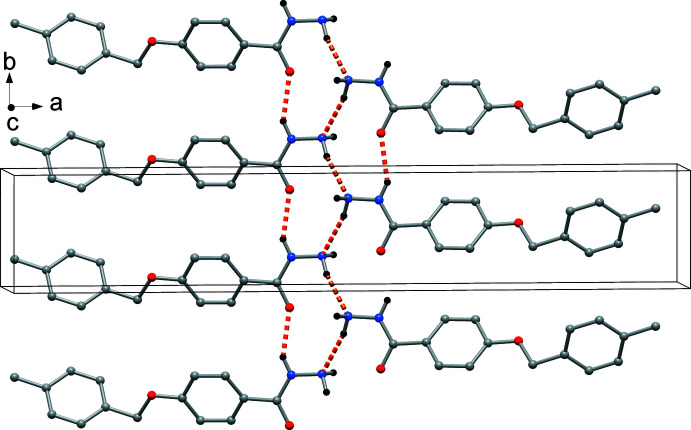
The rows built by N—H⋯N and N—H⋯O inter­actions (orange dashed lines) in the crystal structure of (I).

**Figure 5:**
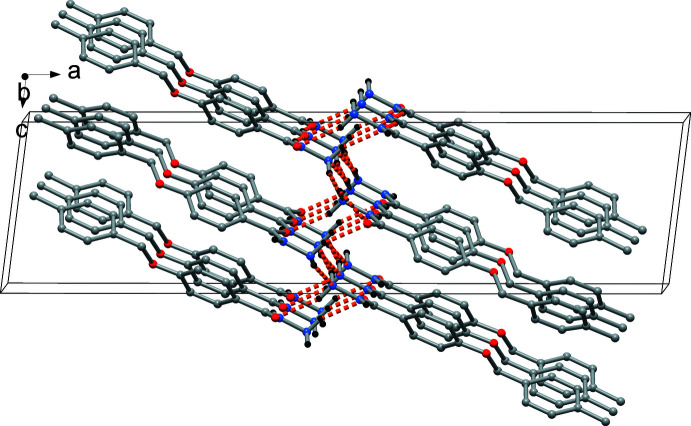
The layered arrangement in the crystal packing of (I) caused by additional N—H⋯O inter­actions.

In (I), these inter­actions are larger because of the higher number of donor hydrogen atoms in the NH—NH_2_ group. Fig. 4 ▸ shows the N1—H1⋯O2^i^ and N2—H2*B*⋯N2^iii^ inter­actions [symmetry codes: (i) *x*, *y* + 1, *z*; (iii) −*x* + 1, *y* − 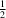 , −*z* − 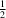 ] connecting rows of oppositely oriented mol­ecules. In addition, N2—H2*A*⋯N2^iii^ inter­actions connect the rows into a layer structure extending parallel to (100) (Fig. 5 ▸).

**Figure 6:**
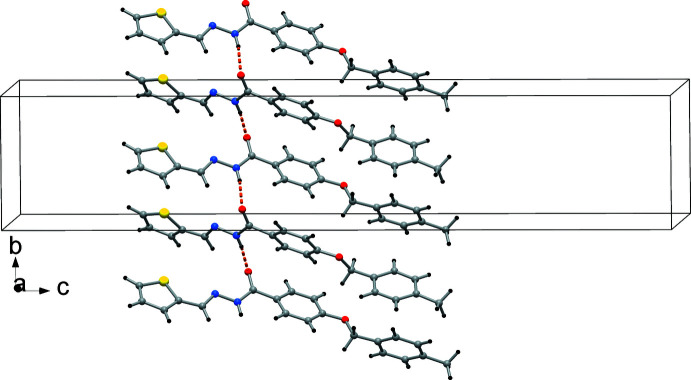
The chain structure in the crystal packing of (II) with indication of N—H⋯O hydrogen bonds (orange dashed lines).

In (II), the number of conventional hydrogen bonds is considerably reduced. The corresponding N1—H1*N*⋯O2^i^ [symmetry code: (i) −*x* + 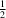 , *y* − 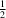 , *z*] inter­actions create an undulating ribbon parallel to [010], as displayed in Fig. 6 ▸.

While π stacking inter­actions in (I) and (II) are insignificant, C—H⋯π-ring inter­actions contribute to the packing in both crystals. These involve the C3—H, C6—H, and C14—H groups with phenyl rings (C2–C7; *Cg*1) and (C9–C14; *Cg*2) in (I) and (II), and the thio­phene ring (*Cg*3) in (II). All the H⋯centroid distances are between 2.77–2.90 Å, with C—H⋯π angles of 122–156° (Tables 1 ▸ and 2 ▸). In (II), additional C—H⋯O and C—H⋯S inter­actions are observed (Table 2 ▸).

## 4. Synthesis and crystallization

*Synthesis of compound (I).* A mixture of ethyl-4-[(4-methyl­benz­yl)­oxy] benzoate (1.23 g, 4.55 mmol) and hydrazine hydrate (5.83 g, 22.69 mmol) in absolute ethanol (20 ml) was refluxed for 10 h. After cooling the solution to room temperature, colorless crystals, suitable for X-ray diffraction, were obtained. Yield: 0.82 g, 70%; melting point: 397–398 K;

FT–IR: 1644 ν (C=O_amide_), 3374 ν (N—H); ^1^H NMR (CDCl_3_, 600 MHz): δ = 2.36 (*s*, 3H, –CH_3_), 4.05 (*brs*, 2H, –NH_2_), 5.07 (*s*, 2H, –CH_2_–), 6.99 (*d*, 2H, Ar-H 5,6, *J* = 13.2 Hz), 7.20 (*d*, 2H, Ar-H 10,11, *J* = 11.4 Hz), 7.30 (*d*, 2H, Ar-H 8,9, *J* = 12 Hz), 7.70 (*d*, 2H, Ar-H 3,4, *J* = 10.2 Hz), ppm; ^13^C NMR (CDCl_3_, 600 MHz): 21.3 (C7), 70.1 (C8), 114.8 (C-3,5), 125.08 (C1), 127.73 (C-2′,6′), 128.7 (C-2,6), 129.4 (C-3′,5′), 133.2 (C1), 138.13 (C4), 161.7 (C4), 168.4 (C9) ppm; LC–MS (ESI) *m*/*z*: [*M* + H]^+^. Calculated for C_15_H_16_N_2_O_2_; 257.1283; found 257.1284. The proton at the NH group was missing, likely due to the exchangeable nature of this proton.

*Synthesis of compound (II).* Thio­phene-2-carbaldehyde (0.15 g, 1.21 mmol) was added to an absolute ethano­lic (20 ml) solution of 4-[(4-methyl­benz­yl)­oxy]benzoyl­hydrazine (0.312 g, 1.21 mmol). The resulting mixture was heated and refluxed for 2 h. A white precipitate was obtained, filtered off, and washed several times with hot ethanol, and finally dried over silica gel in a desiccator. A small amount of the compound was dissolved in 25 ml of absolute ethanol and allowed for slow evaporation. Suitable crystals for single-crystal X-ray diffraction were collected after 30 d of keeping the sample solution undisturbed. Yield: 0.86 g, 50%; melting point: 505–506 K.

FT–IR: 1634 ν (C=O_amide_), 3204 ν (N—H), 1607 (C=N_azomethine_); ^1^H NMR (CDCl_3_, 600 MHz): δ = 2.36 (*s*, 3H, –CH_3_), 5.07 (*s*, 2H, –CH_2_–), 7.03 (*d*, 2H, Ar-H 5,6, *J* = 13.2 Hz), 7.20 (*d*, 2H, Ar-H 10,11, *J* = 11.4 Hz), 7.32 (*d*, 2H, Ar-H 8,9, *J* = 12 Hz), 7.39–7.40 (*m*, 1H, CH=N), 8.9 (*s*, 1H, –CONH–) ppm; LC–MS (ESI) *m*/*z*: [*M* + H]^+.^ Calculated for C_20_H_18_N_2_O_2_S; 351.1159; found 351.1162.

We failed to locate the ^1^H NMR signals of Ar-H 3,4 and of thio­phene ring hydrogen atoms, likely due to the poor solubility of the compound in organic solvents*.*

## 5. Refinement

Crystal data, data collection and structure refinement details are summarized in Table 3 ▸. Hydrogen atoms were calculated at geometrical positions and refined as riding [C—H = 0.95–0.99 Å, *U*_iso_(H) = 1.2*U*_eq_(C)], except those of the —NH—NH_2_ (I) and —NH—N= (II) groups, which were detected in difference-Fourier maps and freely refined.

## Supplementary Material

Crystal structure: contains datablock(s) I, II. DOI: 10.1107/S2056989023001354/wm5671sup1.cif

Structure factors: contains datablock(s) I. DOI: 10.1107/S2056989023001354/wm5671Isup2.hkl

Structure factors: contains datablock(s) II. DOI: 10.1107/S2056989023001354/wm5671IIsup3.hkl

Supporting information file. DOI: 10.1107/S2056989023001354/wm5671Isup4.cml

Supporting information file. DOI: 10.1107/S2056989023001354/wm5671IIsup5.cml

CCDC references: 2232079, 2232115

Additional supporting information:  crystallographic information; 3D view; checkCIF report

## Figures and Tables

**Table 1:** Hydrogen-bond geometry (Å, °) for (I) *Cg*1 and *Cg*2 are the centroids of the C2–C7 and C9–C14 rings, respectively.

*D*—H⋯*A*	*D*—H	H⋯*A*	*D*⋯*A*	*D*—H⋯*A*
N1—H1⋯O2^i^	0.872 (18)	2.228 (18)	2.9994 (14)	147.3 (14)
N2—H2A⋯O2^ii^	0.88 (2)	2.21 (2)	3.0598 (17)	160 (2)
N2—H2B⋯N2^iii^	0.94 (2)	2.27 (2)	3.1970 (14)	167 (1)
C3—H3⋯*Cg*1^iv^	0.95	2.86	3.6085 (16)	136
C6—H6⋯*Cg*1^v^	0.95	2.83	3.5688 (16)	135
C11—H11⋯*Cg*2^vi^	0.95	2.90	3.5918 (13)	131
C14—H14⋯*Cg*2^vii^	0.95	2.92	3.6262 (13)	132

Symmetry codes: (i) 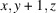 ; (ii) 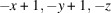 ; (iii) 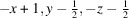 ; (iv) 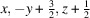 ; (v) 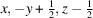 ; (vi) 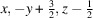 ; (vii) 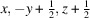 .

**Table 2:** Hydrogen-bond geometry (Å, °) for (II) *Cg*1, *Cg*2 and *Cg*3 are the centroids of the C2–C7, C9–C14 and thio­phene rings, respectively.

*D*—H⋯*A*	*D*—H	H⋯*A*	*D*⋯*A*	*D*—H⋯*A*
N1—H1*N*⋯O2^i^	0.87 (4)	2.04 (4)	2.899 (4)	170 (4)
C10—H10⋯O1^ii^	0.95	2.62	3.409 (4)	140
C16—H16⋯O2^i^	0.95	2.49	3.316 (5)	146
C18—H18⋯S1^iii^	0.95	3.00	3.938 (4)	170
C19—H19⋯O2^iv^	0.95	2.65	3.319 (5)	128
C19—H19⋯O2^iv^	0.95	2.65	3.319 (5)	128
C3—H3⋯*Cg*2^v^	0.95	2.77	3.540 (4)	138
C6—H6⋯*Cg*1^vi^	0.95	2.80	3.593 (4)	141
C14—H14⋯*Cg*2^i^	0.95	2.90	3.504 (4)	122
C18—H18⋯*Cg*3^iii^	0.95	2.92	3.803 (4)	156

Symmetry codes: (i) 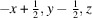 ; (ii) 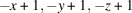 ; (iii) 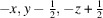 ; (iv) 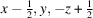 ; (v) 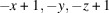 ; (vi) 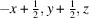 .

**Table 3:** Experimental details

	(I)	(II)
Crystal data		
Chemical formula	C_15_H_16_N_2_O_2_	C_20_H_18_N_2_O_2_S
*M*_r_	256.30	350.42
Crystal system, space group	Monoclinic, *P*2_1_/*c*	Orthorhombic, *P* *b* *c* *a*
Temperature (K)	173	173
*a*, *b*, *c* (Å)	30.7086 (14), 5.2471 (3), 8.0359 (4)	11.3725 (8), 7.8492 (5), 39.286 (2)
α, β, γ (°)	90, 97.471 (7), 90	90, 90, 90
*V* (Å^3^)	1283.85 (11)	3506.8 (4)
*Z*	4	8
Radiation type	Mo *K*α	Mo *K*α
μ (mm^−1^)	0.09	0.20
Crystal size (mm)	0.17 × 0.16 × 0.13	0.26 × 0.08 × 0.01

Data collection		
Diffractometer	Rigaku R-AXIS RAPID	Rigaku R-AXIS RAPID
Absorption correction	Multi-scan (*ABSCOR*; Higashi, 1995 ▸)	Multi-scan (*ABSCOR*; Higashi, 1995 ▸)
*T*_min_, *T*_max_	0.739, 0.988	0.646, 0.998
No. of measured, independent and observed [*I* > 2σ(*I*)] reflections	9727, 2889, 2515	31564, 4006, 2203
*R*_int_	0.024	0.162
(sin θ/λ)_max_ (Å^−1^)	0.648	0.649

Refinement		
*R*[*F* ^2^ > 2σ(*F* ^2^)], *wR*(*F* ^2^), *S*	0.044, 0.126, 1.04	0.078, 0.201, 1.02
No. of reflections	2889	4006
No. of parameters	182	230
H-atom treatment	H atoms treated by a mixture of independent and constrained refinement	H atoms treated by a mixture of independent and constrained refinement
Δρ_max_, Δρ_min_ (e Å^−3^)	0.36, −0.25	0.28, −0.46

Computer programs: *RAPID-AUTO* (Rigaku, 2018 ▸), *SHELXT* (Sheldrick, 2015*a* ▸), *SHELXL* (Sheldrick, 2015*b* ▸), *DIAMOND* (Brandenburg, 1999 ▸) and *WinGX* publication routines (Farrugia, 2012 ▸).

## supplementary crystallographic information

### 4-[(4-Methylbenzyl)oxy]benzohydrazide (I) Crystal data

**Table:**

C_15_H_16_N_2_O_2_	*F*(000) = 544
*M**_r_* = 256.30	*D*_x_ = 1.326 Mg m^−^^3^
Monoclinic, *P*2_1_/*c*	Mo *K*α radiation, λ = 0.71075 Å
*a* = 30.7086 (14) Å	Cell parameters from 8347 reflections
*b* = 5.2471 (3) Å	θ = 2.0–27.4°
*c* = 8.0359 (4) Å	µ = 0.09 mm^−^^1^
β = 97.471 (7)°	*T* = 173 K
*V* = 1283.85 (11) Å^3^	Prism, colorless
*Z* = 4	0.17 × 0.16 × 0.13 mm

### 4-[(4-Methylbenzyl)oxy]benzohydrazide (I) Data collection

**Table:**

Rigaku R-AXIS RAPID diffractometer	2515 reflections with *I* > 2σ(*I*)
Detector resolution: 10.000 pixels mm^-1^	*R*_int_ = 0.024
ω scans	θ_max_ = 27.4°, θ_min_ = 2.7°
Absorption correction: multi-scan (ABSCOR; Higashi, 1995)	*h* = −39→39
*T*_min_ = 0.739, *T*_max_ = 0.988	*k* = −6→6
9727 measured reflections	*l* = −7→10
2889 independent reflections	

### 4-[(4-Methylbenzyl)oxy]benzohydrazide (I) Refinement

**Table:**

Refinement on *F*^2^	0 restraints
Least-squares matrix: full	Hydrogen site location: mixed
*R*[*F*^2^ > 2σ(*F*^2^)] = 0.044	H atoms treated by a mixture of independent and constrained refinement
*wR*(*F*^2^) = 0.126	*w* = 1/[σ^2^(*F*_o_^2^) + (0.0685*P*)^2^ + 0.437*P*] where *P* = (*F*_o_^2^ + 2*F*_c_^2^)/3
*S* = 1.04	(Δ/σ)_max_ = 0.001
2889 reflections	Δρ_max_ = 0.36 e Å^−^^3^
182 parameters	Δρ_min_ = −0.25 e Å^−^^3^

### 4-[(4-Methylbenzyl)oxy]benzohydrazide (I) Special details

**Table:**

Geometry. All esds (except the esd in the dihedral angle between two l.s. planes) are estimated using the full covariance matrix. The cell esds are taken into account individually in the estimation of esds in distances, angles and torsion angles; correlations between esds in cell parameters are only used when they are defined by crystal symmetry. An approximate (isotropic) treatment of cell esds is used for estimating esds involving l.s. planes.

### 4-[(4-Methylbenzyl)oxy]benzohydrazide (I) Fractional atomic coordinates and isotropic or equivalent isotropic displacement parameters (Å^2^)

**Table:**

	*x*	*y*	*z*	*U*_iso_*/*U*_eq_	
O1	0.76070 (3)	0.52411 (17)	0.25819 (12)	0.0286 (2)	
O2	0.56320 (3)	0.27730 (17)	−0.03945 (13)	0.0313 (2)	
N1	0.56104 (3)	0.7071 (2)	−0.06599 (15)	0.0269 (3)	
H1	0.5729 (5)	0.857 (3)	−0.046 (2)	0.032*	
N2	0.51729 (4)	0.7187 (2)	−0.14851 (18)	0.0314 (3)	
H2a	0.4990 (6)	0.724 (3)	−0.072 (3)	0.038*	
H2b	0.5119 (6)	0.568 (4)	−0.212 (2)	0.038*	
C1	0.95555 (5)	0.6618 (4)	0.6481 (2)	0.0471 (4)	
H1A	0.970910	0.734626	0.559722	0.057*	
H1B	0.972202	0.515957	0.699105	0.057*	
H1C	0.952889	0.791252	0.734154	0.057*	
C2	0.91027 (4)	0.5748 (3)	0.57322 (17)	0.0316 (3)	
C3	0.87328 (5)	0.7144 (3)	0.60247 (18)	0.0317 (3)	
H3	0.876869	0.864907	0.668564	0.038*	
C4	0.83138 (4)	0.6368 (3)	0.53651 (18)	0.0295 (3)	
H4	0.806674	0.735051	0.557333	0.035*	
C5	0.82520 (4)	0.4160 (2)	0.44003 (16)	0.0253 (3)	
C6	0.86196 (5)	0.2773 (3)	0.40966 (18)	0.0311 (3)	
H6	0.858381	0.127510	0.342843	0.037*	
C7	0.90393 (4)	0.3557 (3)	0.47622 (19)	0.0343 (3)	
H7	0.928632	0.257732	0.454939	0.041*	
C8	0.77962 (4)	0.3300 (2)	0.37186 (17)	0.0271 (3)	
H8A	0.780838	0.165724	0.312103	0.032*	
H8B	0.761665	0.306844	0.464516	0.032*	
C9	0.71691 (4)	0.5041 (2)	0.19621 (15)	0.0225 (3)	
C10	0.70076 (4)	0.6971 (2)	0.08495 (17)	0.0249 (3)	
H10	0.719868	0.827904	0.056518	0.030*	
C11	0.65699 (4)	0.6983 (2)	0.01604 (16)	0.0244 (3)	
H11	0.646167	0.830898	−0.058621	0.029*	
C12	0.62868 (4)	0.5054 (2)	0.05582 (15)	0.0216 (3)	
C13	0.64525 (4)	0.3131 (2)	0.16527 (16)	0.0234 (3)	
H13	0.626219	0.180748	0.191960	0.028*	
C14	0.68913 (4)	0.3099 (2)	0.23678 (16)	0.0239 (3)	
H14	0.699909	0.177669	0.311821	0.029*	
C15	0.58137 (4)	0.4879 (2)	−0.01986 (16)	0.0232 (3)	

### 4-[(4-Methylbenzyl)oxy]benzohydrazide (I) Atomic displacement parameters (Å^2^)

**Table:**

	*U*^11^	*U*^22^	*U*^33^	*U*^12^	*U*^13^	*U*^23^
O1	0.0213 (4)	0.0294 (5)	0.0335 (5)	−0.0023 (3)	−0.0020 (4)	0.0092 (4)
O2	0.0258 (5)	0.0205 (5)	0.0454 (6)	−0.0024 (3)	−0.0031 (4)	−0.0008 (4)
N1	0.0224 (5)	0.0201 (5)	0.0363 (6)	−0.0013 (4)	−0.0040 (4)	0.0005 (4)
N2	0.0222 (5)	0.0246 (6)	0.0448 (7)	0.0003 (4)	−0.0063 (5)	0.0026 (5)
C1	0.0287 (7)	0.0612 (11)	0.0487 (10)	−0.0065 (7)	−0.0055 (6)	0.0012 (8)
C2	0.0262 (6)	0.0368 (7)	0.0303 (7)	−0.0019 (5)	−0.0021 (5)	0.0065 (6)
C3	0.0335 (7)	0.0293 (7)	0.0314 (7)	−0.0032 (5)	0.0005 (5)	−0.0024 (5)
C4	0.0266 (6)	0.0286 (6)	0.0330 (7)	0.0036 (5)	0.0032 (5)	−0.0013 (5)
C5	0.0240 (6)	0.0260 (6)	0.0250 (6)	0.0006 (5)	0.0002 (5)	0.0045 (5)
C6	0.0307 (7)	0.0278 (6)	0.0339 (7)	0.0043 (5)	0.0005 (5)	−0.0019 (5)
C7	0.0249 (6)	0.0393 (7)	0.0384 (8)	0.0078 (6)	0.0028 (5)	0.0020 (6)
C8	0.0252 (6)	0.0256 (6)	0.0291 (6)	0.0003 (5)	−0.0014 (5)	0.0048 (5)
C9	0.0216 (5)	0.0228 (6)	0.0228 (6)	0.0004 (4)	0.0016 (4)	−0.0014 (4)
C10	0.0242 (6)	0.0215 (6)	0.0290 (7)	−0.0036 (4)	0.0031 (5)	0.0030 (5)
C11	0.0259 (6)	0.0200 (6)	0.0268 (6)	0.0011 (5)	0.0019 (5)	0.0035 (4)
C12	0.0217 (6)	0.0191 (5)	0.0237 (6)	0.0009 (4)	0.0018 (4)	−0.0030 (4)
C13	0.0239 (6)	0.0196 (5)	0.0266 (6)	−0.0025 (4)	0.0030 (5)	0.0006 (4)
C14	0.0259 (6)	0.0204 (6)	0.0250 (6)	0.0009 (4)	0.0015 (5)	0.0025 (4)
C15	0.0235 (6)	0.0215 (6)	0.0242 (6)	−0.0002 (4)	0.0017 (5)	−0.0007 (4)

### 4-[(4-Methylbenzyl)oxy]benzohydrazide (I) Geometric parameters (Å, º)

**Table:**

O1—C9	1.3758 (14)	C5—C6	1.3913 (18)
O1—C8	1.4391 (15)	C5—C8	1.5043 (17)
O2—C15	1.2388 (15)	C6—C7	1.3918 (19)
N1—C15	1.3380 (15)	C6—H6	0.9500
N1—N2	1.4200 (15)	C7—H7	0.9500
N1—H1	0.872 (18)	C8—H8A	0.9900
N2—H2A	0.88 (2)	C8—H8B	0.9900
N2—H2B	0.94 (2)	C9—C14	1.3945 (17)
C1—C2	1.5123 (19)	C9—C10	1.3986 (17)
C1—H1A	0.9800	C10—C11	1.3853 (17)
C1—H1B	0.9800	C10—H10	0.9500
C1—H1C	0.9800	C11—C12	1.3982 (16)
C2—C7	1.388 (2)	C11—H11	0.9500
C2—C3	1.397 (2)	C12—C13	1.3908 (17)
C3—C4	1.3876 (18)	C12—C15	1.5031 (16)
C3—H3	0.9500	C13—C14	1.3946 (16)
C4—C5	1.3930 (18)	C13—H13	0.9500
C4—H4	0.9500	C14—H14	0.9500
			
C9—O1—C8	118.08 (9)	C2—C7—H7	119.5
C15—N1—N2	123.03 (10)	C6—C7—H7	119.5
C15—N1—H1	123.6 (11)	O1—C8—C5	107.39 (10)
N2—N1—H1	113.3 (11)	O1—C8—H8A	110.2
N1—N2—H2A	108.9 (12)	C5—C8—H8A	110.2
N1—N2—H2B	107.8 (10)	O1—C8—H8B	110.2
H2A—N2—H2B	108.7 (16)	C5—C8—H8B	110.2
C2—C1—H1A	109.5	H8A—C8—H8B	108.5
C2—C1—H1B	109.5	O1—C9—C14	124.75 (11)
H1A—C1—H1B	109.5	O1—C9—C10	115.14 (10)
C2—C1—H1C	109.5	C14—C9—C10	120.11 (11)
H1A—C1—H1C	109.5	C11—C10—C9	120.26 (11)
H1B—C1—H1C	109.5	C11—C10—H10	119.9
C7—C2—C3	118.10 (12)	C9—C10—H10	119.9
C7—C2—C1	121.88 (13)	C10—C11—C12	120.33 (11)
C3—C2—C1	120.02 (14)	C10—C11—H11	119.8
C4—C3—C2	121.04 (13)	C12—C11—H11	119.8
C4—C3—H3	119.5	C13—C12—C11	118.86 (11)
C2—C3—H3	119.5	C13—C12—C15	117.80 (10)
C3—C4—C5	120.61 (12)	C11—C12—C15	123.28 (11)
C3—C4—H4	119.7	C12—C13—C14	121.59 (11)
C5—C4—H4	119.7	C12—C13—H13	119.2
C6—C5—C4	118.54 (12)	C14—C13—H13	119.2
C6—C5—C8	121.12 (12)	C13—C14—C9	118.85 (11)
C4—C5—C8	120.33 (11)	C13—C14—H14	120.6
C5—C6—C7	120.65 (13)	C9—C14—H14	120.6
C5—C6—H6	119.7	O2—C15—N1	123.06 (11)
C7—C6—H6	119.7	O2—C15—C12	120.11 (10)
C2—C7—C6	121.05 (12)	N1—C15—C12	116.83 (10)
			
C7—C2—C3—C4	0.0 (2)	C14—C9—C10—C11	0.79 (19)
C1—C2—C3—C4	−179.31 (14)	C9—C10—C11—C12	−0.60 (19)
C2—C3—C4—C5	0.4 (2)	C10—C11—C12—C13	−0.06 (18)
C3—C4—C5—C6	−0.8 (2)	C10—C11—C12—C15	−177.22 (11)
C3—C4—C5—C8	178.74 (12)	C11—C12—C13—C14	0.55 (18)
C4—C5—C6—C7	0.9 (2)	C15—C12—C13—C14	177.86 (11)
C8—C5—C6—C7	−178.67 (13)	C12—C13—C14—C9	−0.36 (18)
C3—C2—C7—C6	0.1 (2)	O1—C9—C14—C13	179.74 (11)
C1—C2—C7—C6	179.37 (14)	C10—C9—C14—C13	−0.31 (18)
C5—C6—C7—C2	−0.5 (2)	N2—N1—C15—O2	−2.9 (2)
C9—O1—C8—C5	−171.75 (10)	N2—N1—C15—C12	175.97 (12)
C6—C5—C8—O1	−117.28 (13)	C13—C12—C15—O2	−27.44 (17)
C4—C5—C8—O1	63.20 (16)	C11—C12—C15—O2	149.75 (13)
C8—O1—C9—C14	0.69 (18)	C13—C12—C15—N1	153.70 (12)
C8—O1—C9—C10	−179.27 (11)	C11—C12—C15—N1	−29.12 (17)
O1—C9—C10—C11	−179.25 (11)		

### 4-[(4-Methylbenzyl)oxy]benzohydrazide (I) Hydrogen-bond geometry (Å, º)

*Cg*1 and *Cg*2 are the centroids of the C2–C7 and C9–C14 rings, respectively.

**Table:**

*D*—H···*A*	*D*—H	H···*A*	*D*···*A*	*D*—H···*A*
N1—H1···O2^i^	0.872 (18)	2.228 (18)	2.9994 (14)	147.3 (14)
N2—H2A···O2^ii^	0.88 (2)	2.21 (2)	3.0598 (17)	160 (2)
N2—H2B···N2^iii^	0.94 (2)	2.27 (2)	3.1970 (14)	167 (1)
C3—H3···*Cg*1^iv^	0.95	2.86	3.6085 (16)	136
C6—H6···*Cg*1^v^	0.95	2.83	3.5688 (16)	135
C11—H11···*Cg*2^vi^	0.95	2.90	3.5918 (13)	131
C14—H14···*Cg*2^vii^	0.95	2.92	3.6262 (13)	132

Symmetry codes: (i) *x*, *y*+1, *z*; (ii) −*x*+1, −*y*+1, −*z*; (iii) −*x*+1, *y*−1/2, −*z*−1/2; (iv) *x*, −*y*+3/2, *z*+1/2; (v) *x*, −*y*+1/2, *z*−1/2; (vi) *x*, −*y*+3/2, *z*−1/2; (vii) *x*, −*y*+1/2, *z*+1/2.

### 4-[(4-Methylbenzyl)oxy]-N'-[(thiophen-2-yl)methylidene]benzohydrazide (II) Crystal data

**Table:**

C_20_H_18_N_2_O_2_S	*D*_x_ = 1.327 Mg m^−^^3^
*M**_r_* = 350.42	Mo *K*α radiation, λ = 0.71075 Å
Orthorhombic, *P**b**c**a*	Cell parameters from 12507 reflections
*a* = 11.3725 (8) Å	θ = 1.9–27.4°
*b* = 7.8492 (5) Å	µ = 0.20 mm^−^^1^
*c* = 39.286 (2) Å	*T* = 173 K
*V* = 3506.8 (4) Å^3^	Plate, colorless
*Z* = 8	0.26 × 0.08 × 0.01 mm
*F*(000) = 1472	

### 4-[(4-Methylbenzyl)oxy]-N'-[(thiophen-2-yl)methylidene]benzohydrazide (II) Data collection

**Table:**

Rigaku R-AXIS RAPID diffractometer	2203 reflections with *I* > 2σ(*I*)
Detector resolution: 10.000 pixels mm^-1^	*R*_int_ = 0.162
ω scans	θ_max_ = 27.5°, θ_min_ = 2.1°
Absorption correction: multi-scan (ABSCOR; Higashi, 1995)	*h* = −14→14
*T*_min_ = 0.646, *T*_max_ = 0.998	*k* = −10→10
31564 measured reflections	*l* = −50→50
4006 independent reflections	

### 4-[(4-Methylbenzyl)oxy]-N'-[(thiophen-2-yl)methylidene]benzohydrazide (II) Refinement

**Table:**

Refinement on *F*^2^	0 restraints
Least-squares matrix: full	Hydrogen site location: mixed
*R*[*F*^2^ > 2σ(*F*^2^)] = 0.078	H atoms treated by a mixture of independent and constrained refinement
*wR*(*F*^2^) = 0.201	*w* = 1/[σ^2^(*F*_o_^2^) + (0.0867*P*)^2^ + 2.4194*P*] where *P* = (*F*_o_^2^ + 2*F*_c_^2^)/3
*S* = 1.02	(Δ/σ)_max_ < 0.001
4006 reflections	Δρ_max_ = 0.28 e Å^−^^3^
230 parameters	Δρ_min_ = −0.46 e Å^−^^3^

### 4-[(4-Methylbenzyl)oxy]-N'-[(thiophen-2-yl)methylidene]benzohydrazide (II) Special details

**Table:**

Geometry. All esds (except the esd in the dihedral angle between two l.s. planes) are estimated using the full covariance matrix. The cell esds are taken into account individually in the estimation of esds in distances, angles and torsion angles; correlations between esds in cell parameters are only used when they are defined by crystal symmetry. An approximate (isotropic) treatment of cell esds is used for estimating esds involving l.s. planes.

### 4-[(4-Methylbenzyl)oxy]-N'-[(thiophen-2-yl)methylidene]benzohydrazide (II) Fractional atomic coordinates and isotropic or equivalent isotropic displacement parameters (Å^2^)

**Table:**

	*x*	*y*	*z*	*U*_iso_*/*U*_eq_	
S1	0.22046 (10)	0.59338 (16)	0.23512 (3)	0.0543 (3)	
O1	0.3869 (2)	0.2842 (3)	0.50032 (6)	0.0403 (7)	
O2	0.3905 (2)	0.6420 (3)	0.35634 (6)	0.0364 (6)	
N1	0.2605 (3)	0.4326 (4)	0.34359 (8)	0.0384 (8)	
H1N	0.220 (4)	0.343 (5)	0.3497 (10)	0.046*	
N2	0.2419 (3)	0.4962 (4)	0.31082 (7)	0.0361 (7)	
C1	0.4445 (4)	−0.0531 (6)	0.65013 (10)	0.0586 (12)	
H1A	0.432854	0.036538	0.667172	0.070*	
H1B	0.527637	−0.085807	0.649593	0.070*	
H1C	0.396550	−0.152458	0.656079	0.070*	
C2	0.4081 (3)	0.0126 (5)	0.61544 (9)	0.0399 (9)	
C3	0.4761 (3)	−0.0238 (5)	0.58686 (9)	0.0387 (9)	
H3	0.546925	−0.086739	0.589404	0.046*	
C4	0.4420 (3)	0.0303 (5)	0.55480 (10)	0.0380 (9)	
H4	0.488311	0.001132	0.535548	0.046*	
C5	0.3407 (3)	0.1269 (5)	0.55051 (9)	0.0355 (9)	
C6	0.2739 (3)	0.1669 (5)	0.57909 (9)	0.0428 (10)	
H6	0.204834	0.233913	0.576795	0.051*	
C7	0.3084 (3)	0.1088 (6)	0.61105 (10)	0.0482 (11)	
H7	0.261670	0.136347	0.630331	0.058*	
C8	0.2990 (3)	0.1780 (5)	0.51590 (9)	0.0391 (9)	
H8A	0.285445	0.075437	0.501774	0.047*	
H8B	0.223934	0.241183	0.517765	0.047*	
C9	0.3674 (3)	0.3338 (5)	0.46694 (9)	0.0345 (8)	
C10	0.4558 (3)	0.4314 (5)	0.45250 (9)	0.0369 (9)	
H10	0.523935	0.459873	0.465289	0.044*	
C11	0.4438 (3)	0.4872 (5)	0.41914 (9)	0.0351 (8)	
H11	0.503537	0.555552	0.409199	0.042*	
C12	0.3447 (3)	0.4438 (4)	0.40007 (9)	0.0318 (8)	
C13	0.2571 (3)	0.3477 (5)	0.41541 (9)	0.0334 (8)	
H13	0.188522	0.319851	0.402784	0.040*	
C14	0.2673 (3)	0.2915 (4)	0.44869 (9)	0.0337 (8)	
H14	0.206828	0.225189	0.458814	0.040*	
C15	0.3346 (3)	0.5139 (5)	0.36493 (9)	0.0321 (8)	
C16	0.1662 (3)	0.4133 (5)	0.29375 (9)	0.0372 (9)	
H16	0.128409	0.318128	0.303940	0.045*	
C17	0.1361 (3)	0.4599 (5)	0.25919 (9)	0.0380 (9)	
C18	0.0398 (3)	0.4010 (5)	0.24049 (9)	0.0402 (9)	
H18	−0.018133	0.325460	0.249223	0.048*	
C19	0.0387 (4)	0.4664 (6)	0.20742 (11)	0.0597 (13)	
H19	−0.019933	0.439397	0.191077	0.072*	
C20	0.1296 (4)	0.5718 (7)	0.20114 (11)	0.0602 (13)	
H20	0.141558	0.627988	0.179994	0.072*	

### 4-[(4-Methylbenzyl)oxy]-N'-[(thiophen-2-yl)methylidene]benzohydrazide (II) Atomic displacement parameters (Å^2^)

**Table:**

	*U*^11^	*U*^22^	*U*^33^	*U*^12^	*U*^13^	*U*^23^
S1	0.0499 (7)	0.0616 (7)	0.0514 (7)	−0.0076 (6)	0.0008 (5)	0.0057 (6)
O1	0.0346 (15)	0.0494 (16)	0.0369 (14)	−0.0071 (12)	−0.0077 (11)	0.0036 (13)
O2	0.0283 (13)	0.0410 (14)	0.0400 (15)	−0.0030 (12)	0.0020 (11)	0.0010 (12)
N1	0.0334 (18)	0.0432 (19)	0.0384 (18)	−0.0054 (15)	−0.0072 (14)	0.0057 (16)
N2	0.0288 (16)	0.0430 (17)	0.0365 (16)	0.0033 (14)	−0.0040 (13)	0.0031 (15)
C1	0.046 (3)	0.082 (3)	0.048 (3)	−0.003 (2)	−0.009 (2)	0.016 (2)
C2	0.032 (2)	0.046 (2)	0.042 (2)	−0.0084 (18)	−0.0063 (16)	0.0045 (19)
C3	0.0222 (18)	0.044 (2)	0.050 (2)	−0.0003 (16)	−0.0057 (16)	0.0036 (19)
C4	0.0222 (18)	0.047 (2)	0.045 (2)	−0.0016 (17)	0.0027 (16)	−0.0018 (18)
C5	0.0261 (18)	0.039 (2)	0.041 (2)	−0.0020 (16)	−0.0036 (16)	0.0047 (18)
C6	0.026 (2)	0.055 (2)	0.047 (2)	0.0075 (18)	−0.0027 (16)	−0.004 (2)
C7	0.033 (2)	0.069 (3)	0.043 (2)	0.005 (2)	0.0003 (17)	−0.004 (2)
C8	0.030 (2)	0.044 (2)	0.043 (2)	−0.0070 (17)	−0.0024 (16)	0.0029 (19)
C9	0.027 (2)	0.039 (2)	0.038 (2)	0.0007 (15)	−0.0028 (15)	0.0005 (17)
C10	0.0260 (19)	0.044 (2)	0.041 (2)	−0.0024 (16)	−0.0056 (15)	−0.0027 (18)
C11	0.0263 (19)	0.037 (2)	0.042 (2)	−0.0003 (16)	0.0008 (15)	0.0000 (17)
C12	0.0254 (18)	0.034 (2)	0.036 (2)	0.0053 (15)	0.0001 (14)	−0.0019 (16)
C13	0.0244 (18)	0.0373 (19)	0.039 (2)	0.0027 (15)	−0.0039 (15)	−0.0037 (17)
C14	0.0267 (19)	0.038 (2)	0.0365 (19)	−0.0033 (16)	0.0010 (15)	0.0003 (17)
C15	0.0223 (18)	0.038 (2)	0.036 (2)	0.0077 (16)	0.0047 (14)	−0.0011 (17)
C16	0.030 (2)	0.039 (2)	0.042 (2)	0.0035 (17)	−0.0002 (16)	0.0043 (18)
C17	0.030 (2)	0.045 (2)	0.038 (2)	0.0058 (17)	−0.0012 (15)	0.0025 (18)
C18	0.0267 (19)	0.053 (2)	0.041 (2)	−0.0067 (18)	−0.0040 (15)	0.0010 (19)
C19	0.044 (3)	0.082 (3)	0.054 (3)	0.013 (3)	−0.020 (2)	−0.004 (3)
C20	0.066 (3)	0.074 (3)	0.040 (2)	0.014 (3)	−0.001 (2)	0.010 (2)

### 4-[(4-Methylbenzyl)oxy]-N'-[(thiophen-2-yl)methylidene]benzohydrazide (II) Geometric parameters (Å, º)

**Table:**

S1—C20	1.697 (5)	C7—H7	0.9500
S1—C17	1.707 (4)	C8—H8A	0.9900
O1—C9	1.386 (4)	C8—H8B	0.9900
O1—C8	1.438 (4)	C9—C14	1.386 (5)
O2—C15	1.236 (4)	C9—C10	1.386 (5)
N1—C15	1.349 (5)	C10—C11	1.389 (5)
N1—N2	1.397 (4)	C10—H10	0.9500
N1—H1N	0.87 (4)	C11—C12	1.395 (5)
N2—C16	1.271 (5)	C11—H11	0.9500
C1—C2	1.515 (5)	C12—C13	1.387 (5)
C1—H1A	0.9800	C12—C15	1.491 (5)
C1—H1B	0.9800	C13—C14	1.385 (5)
C1—H1C	0.9800	C13—H13	0.9500
C2—C7	1.373 (5)	C14—H14	0.9500
C2—C3	1.393 (5)	C16—C17	1.447 (5)
C3—C4	1.385 (5)	C16—H16	0.9500
C3—H3	0.9500	C17—C18	1.398 (5)
C4—C5	1.389 (5)	C18—C19	1.397 (6)
C4—H4	0.9500	C18—H18	0.9500
C5—C6	1.391 (5)	C19—C20	1.347 (7)
C5—C8	1.495 (5)	C19—H19	0.9500
C6—C7	1.392 (5)	C20—H20	0.9500
C6—H6	0.9500		
			
C20—S1—C17	91.9 (2)	C14—C9—C10	121.1 (3)
C9—O1—C8	117.0 (3)	C14—C9—O1	123.6 (3)
C15—N1—N2	119.9 (3)	C10—C9—O1	115.3 (3)
C15—N1—H1N	122 (3)	C9—C10—C11	119.3 (3)
N2—N1—H1N	118 (3)	C9—C10—H10	120.4
C16—N2—N1	114.0 (3)	C11—C10—H10	120.4
C2—C1—H1A	109.5	C10—C11—C12	120.6 (3)
C2—C1—H1B	109.5	C10—C11—H11	119.7
H1A—C1—H1B	109.5	C12—C11—H11	119.7
C2—C1—H1C	109.5	C13—C12—C11	118.7 (3)
H1A—C1—H1C	109.5	C13—C12—C15	123.2 (3)
H1B—C1—H1C	109.5	C11—C12—C15	118.0 (3)
C7—C2—C3	118.1 (3)	C14—C13—C12	121.6 (3)
C7—C2—C1	121.7 (4)	C14—C13—H13	119.2
C3—C2—C1	120.2 (4)	C12—C13—H13	119.2
C4—C3—C2	121.0 (3)	C13—C14—C9	118.8 (3)
C4—C3—H3	119.5	C13—C14—H14	120.6
C2—C3—H3	119.5	C9—C14—H14	120.6
C3—C4—C5	120.7 (3)	O2—C15—N1	122.4 (3)
C3—C4—H4	119.7	O2—C15—C12	120.9 (3)
C5—C4—H4	119.7	N1—C15—C12	116.7 (3)
C4—C5—C6	118.5 (3)	N2—C16—C17	121.7 (4)
C4—C5—C8	121.3 (3)	N2—C16—H16	119.1
C6—C5—C8	120.0 (3)	C17—C16—H16	119.1
C5—C6—C7	120.0 (4)	C18—C17—C16	126.5 (4)
C5—C6—H6	120.0	C18—C17—S1	110.6 (3)
C7—C6—H6	120.0	C16—C17—S1	122.8 (3)
C2—C7—C6	121.7 (4)	C19—C18—C17	111.9 (4)
C2—C7—H7	119.1	C19—C18—H18	124.0
C6—C7—H7	119.1	C17—C18—H18	124.0
O1—C8—C5	108.8 (3)	C20—C19—C18	113.0 (4)
O1—C8—H8A	109.9	C20—C19—H19	123.5
C5—C8—H8A	109.9	C18—C19—H19	123.5
O1—C8—H8B	109.9	C19—C20—S1	112.6 (3)
C5—C8—H8B	109.9	C19—C20—H20	123.7
H8A—C8—H8B	108.3	S1—C20—H20	123.7
			
C15—N1—N2—C16	−176.9 (3)	C11—C12—C13—C14	−1.5 (5)
C7—C2—C3—C4	−2.2 (6)	C15—C12—C13—C14	−176.9 (3)
C1—C2—C3—C4	177.8 (4)	C12—C13—C14—C9	0.4 (5)
C2—C3—C4—C5	1.9 (6)	C10—C9—C14—C13	0.5 (5)
C3—C4—C5—C6	−0.4 (5)	O1—C9—C14—C13	−179.9 (3)
C3—C4—C5—C8	−176.5 (3)	N2—N1—C15—O2	−2.1 (5)
C4—C5—C6—C7	−0.7 (6)	N2—N1—C15—C12	176.6 (3)
C8—C5—C6—C7	175.4 (4)	C13—C12—C15—O2	154.6 (3)
C3—C2—C7—C6	1.0 (6)	C11—C12—C15—O2	−20.9 (5)
C1—C2—C7—C6	−179.0 (4)	C13—C12—C15—N1	−24.2 (5)
C5—C6—C7—C2	0.5 (6)	C11—C12—C15—N1	160.3 (3)
C9—O1—C8—C5	175.3 (3)	N1—N2—C16—C17	−179.5 (3)
C4—C5—C8—O1	−62.3 (5)	N2—C16—C17—C18	−166.6 (4)
C6—C5—C8—O1	121.7 (4)	N2—C16—C17—S1	15.7 (5)
C8—O1—C9—C14	2.3 (5)	C20—S1—C17—C18	0.2 (3)
C8—O1—C9—C10	−178.1 (3)	C20—S1—C17—C16	178.2 (3)
C14—C9—C10—C11	−0.2 (5)	C16—C17—C18—C19	−177.8 (4)
O1—C9—C10—C11	−179.9 (3)	S1—C17—C18—C19	0.1 (4)
C9—C10—C11—C12	−0.9 (5)	C17—C18—C19—C20	−0.5 (6)
C10—C11—C12—C13	1.7 (5)	C18—C19—C20—S1	0.6 (5)
C10—C11—C12—C15	177.4 (3)	C17—S1—C20—C19	−0.5 (4)

### 4-[(4-Methylbenzyl)oxy]-N'-[(thiophen-2-yl)methylidene]benzohydrazide (II) Hydrogen-bond geometry (Å, º)

*Cg*1, *Cg*2 and *Cg*3 are the centroids of the C2–C7, C9–C14 and thiophene rings, respectively.

**Table:**

*D*—H···*A*	*D*—H	H···*A*	*D*···*A*	*D*—H···*A*
N1—H1*N*···O2^i^	0.87 (4)	2.04 (4)	2.899 (4)	170 (4)
C10—H10···O1^ii^	0.95	2.62	3.409 (4)	140
C16—H16···O2^i^	0.95	2.49	3.316 (5)	146
C18—H18···S1^iii^	0.95	3.00	3.938 (4)	170
C19—H19···O2^iv^	0.95	2.65	3.319 (5)	128
C19—H19···O2^iv^	0.95	2.65	3.319 (5)	128
C3—H3···*Cg*2^v^	0.95	2.77	3.540 (4)	138
C6—H6···*Cg*1^vi^	0.95	2.80	3.593 (4)	141
C14—H14···*Cg*2^i^	0.95	2.90	3.504 (4)	122
C18—H18···*Cg*3^iii^	0.95	2.92	3.803 (4)	156

Symmetry codes: (i) −*x*+1/2, *y*−1/2, *z*; (ii) −*x*+1, −*y*+1, −*z*+1; (iii) −*x*, *y*−1/2, −*z*+1/2; (iv) *x*−1/2, *y*, −*z*+1/2; (v) −*x*+1, −*y*, −*z*+1; (vi) −*x*+1/2, *y*+1/2, *z*.
